# Comparing Interviewer-Administered and Web-Based Food Frequency Questionnaires to Predict Energy Requirements in Adults

**DOI:** 10.3390/nu10091292

**Published:** 2018-09-12

**Authors:** Didier Brassard, Simone Lemieux, Amélie Charest, Annie Lapointe, Patrick Couture, Marie-Ève Labonté, Benoît Lamarche

**Affiliations:** 1 Institute of Nutrition and Functional Foods (INAF), Laval University, Quebec City, QC G1V 0A6, Canada; didier.brassard.1@ulaval.ca (D.B.); simone.lemieux@fsaa.ulaval.ca (S.L.); Amelie.Charest@fsaa.ulaval.ca (A.C.); Annie.Lapointe@fsaa.ulaval.ca (A.L.); patrick.couture@crchudequebec.ulaval.ca (P.C.); Marie-Eve.Labonte@fsaa.ulaval.ca (M.-È.L.); 2 School of Nutrition, Laval University, Quebec City, QC G1V 0A6, Canada

**Keywords:** food frequency questionnaire, dietary assessment, web, under-reporting, over-reporting, energy intake

## Abstract

Traditional food frequency questionnaires (FFQs) are influenced by systematic error, but web-based FFQ (WEB-FFQs) may mitigate this source of error. The objective of this study was to compare the accuracy of interview-based and web-based FFQs to assess energy requirements (mERs). The mER was measured in a series of controlled feeding trials in which participants daily received all foods and caloric drinks to maintain stable body weight over 4 to 6 weeks. FFQs assessing dietary intakes and hence mean energy intake were either interviewer-administered by a registered dietitian (IA-FFQ, *n* = 127; control method) or self-administered using a web-based platform (WEB-FFQ, *n* = 200; test method), on a single occasion. Comparison between self-reported energy intake and mER revealed significant under-reporting with the IA-FFQ (−9.5%; 95% CI, −12.7 to −6.1) and with the WEB-FFQ (−11.0%; 95% CI, −15.4 to −6.4), but to a similar extent between FFQs (*p* = 0.62). However, a greater proportion of individuals were considered as accurate reporters of energy intake using the IA-FFQ compared with the WEB-FFQ (67.7% vs. 48.0%, respectively), while the prevalence of over-reporting was lower with the IA-FFQ than with the WEB-FFQ (6.3% vs. 17.5%, respectively). These results suggest less accurate prediction of true energy intake by a self-administered WEB-FFQ than with an IA-FFQ.

## 1. Introduction

Dietary assessment is central to nutritional epidemiology, which forms the basis of dietary guidelines [1,2]. Twenty-four-hour recalls (24HRs) and food frequency questionnaires (FFQs) are common instruments to collect self-reported dietary intakes [3]. However, the validity of self-reported data obtained via such memory-based dietary assessment methods, and hence the whole value of nutrition epidemiology, is being challenged based on their purported inability to correctly reflect true food and nutrient consumption [4,5,6,7]. However, others have argued that despite recognized limitations, relying on self-reported dietary intake data in epidemiological studies has been instrumental in developing impactful dietary guidelines and recommendations over the years [1,2,7,8,9,10]. One of the fundamental issues in this heated debate relates to whether 24HRs and FFQs can measure true energy intake, due among other factors to significant random and systematic errors [1,11,12,13,14].

New methods of dietary assessment using recent technologies are being developed and examined [15,16,17] and there is growing interest in the ability of web-based alternatives to improve the efficiency of data collection. Web-based tools increase the efficiency of the data processing; they can be completed at any time or location, they offer unique advantage regarding portion size presentation and food recognition, and they are cost-effective [16,18]. However, the extent to which web-based delivery methods may mitigate some of the errors seen with more traditional interview-administered (IA) methods such as FFQs remains uncertain. Previous data have suggested that web-based 24HRs may be less prone to social desirability bias compared with IA tools [18,19,20]. The use of digital pictures in a web-based 24HR has also been proposed to facilitate portion size estimation compared with an IA-24HR [21]. A recent review of Canadian epidemiological studies reported that web-based dietary assessment instruments have not yet been used [22]. Thus, the value of web tools needs to be examined carefully for robustness, validity and reproducibility before their use can be expanded in large epidemiological studies.

To the best of our knowledge, no study has yet compared the accuracy of an IA-FFQ and self-administered web-based FFQ (WEB-FFQ) to predict an objective measure of energy requirements (mERs). The primary objective of this study was therefore to compare the accuracy of an IA-FFQ and a WEB-FFQ to assess the mER. Our hypothesis was that the WEB-FFQ is more accurate in assessing the mER than the IA-FFQ.

## 2. Materials and Methods 

### 2.1. Study Design and Population

As a secondary analysis, subjects included in this study were participants from a series of nine randomized and fully controlled feeding trials (six published to date) conducted at the Institute of Nutrition and Functional Foods in Quebec City and at the Richardson Centre For Functional Foods and Nutraceuticals in Winnipeg from 2008 to 2017. All trials were devised to test the impact of different diets and nutrients on cardiometabolic risk factors [23,24,25,26,27,28]. Briefly, participants in these trials were between 18 and 65 years of age, were non-smokers, and had no history of cardiovascular disease, type 1 or type 2 diabetes, monogenic dyslipidemia, or uncontrolled endocrine disorder. Participants had to have maintained a stable body weight (within 2.5 kg) for at least 3 months before the onset of the interventions. All trials considered in the present study were conducted in weight-stable participants. All participants gave their informed consent for inclusion before they participated in the trials included in the present study, which were approved by local ethic boards.

### 2.2. Anthropometric Assessment

Body weight, waist and hip circumference were measured according to standardized procedures after a 12-hour overnight fast before and after each intervention period [29]. In addition, body weight was measured continuously throughout all feeding phases, three to five times per week [23,24,25,26,27,28].

### 2.3. Reported Energy Intake (rEI)

The IA- and WEB-FFQs were previously validated for use in French-speaking adults and details have been published elsewhere [15,30]. Briefly, the IA-FFQ is a face-to-face interviewer-administered FFQ designed to reflect dietary intakes of the past 30 days. The questionnaire is based on typical food items available in the province of Quebec with a special focus on components of the Mediterranean diet in a North-American context, which was required for the trials conducted at the time. The IA-FFQ has 91 items and food models were used in the interviews to facilitate portion size estimation. Administration of the IA-FFQ by a registered dietitian took approximately 30–45 min using standardized language across all participants.

The WEB-FFQ is a self-administered web-based questionnaire also designed to reflect dietary intakes over the past 30 days. Participants completed the WEB-FFQ on-site or at home, using Internet. The questionnaire has 136 questions which were based on the Willet FFQ and the previously validated IA-FFQ [30]. Several serving sizes based on the *Supplementation en Vitamines et Mineraux Antioxydants* (SU.VI.MAX) Food atlas [31] were digitally photographed using standardized dinnerware. Participants completed either the IA-FFQ or the WEB-FFQ, once during the run-in period (i.e., 0 to 4 weeks) preceding the first phase of each controlled feeding trial.

### 2.4. Measured Energy Requirement (mER)

Energy expenditure for each participant was first estimated with validated equations [32] and from the results of the IA- or WEB-FFQ prior to undertaking the intervention phases of the trials. During all phases, participants were asked to come to the laboratory of participating centers at least three times a week in order to pick up meals and snacks and for body weight measurement. Participants were instructed to consume all and only the foods and caloric drinks provided. Dietetic technicians prepared all meals and snacks in the metabolic kitchen of participating centers to the nearest 0.1 g. Participants received all foods and caloric drinks on a daily basis under isoenergetic conditions to maintain body weight constant over feeding phases of 4 to 6 weeks. Food provision was adjusted when body weight fluctuated by more than 2 kg over one week or with any major change in reported hunger or fullness. Participants were instructed to maintain their usual physical activity habits.

mER is considered as a valid estimate of true energy expenditure because energy intake during the feeding trials was adjusted constantly to achieve body weight stability [23,24,25,26,27,28]. Furthermore, controlled feeding studies conducted at the Institute of Nutrition and Functional Foods have been previously used to assess the validity of another web-based instrument [33]. Only the first phase of each trial was considered in the present study due to temporal proximity with the completion of either FFQ. The mER was the mean daily total energy provided to each participant during the fourth week of all feeding phases. Compliance with the dietary intervention was assessed using various approaches. Self-reported compliance assessed using checklists was high across all interventions (>98%) with a large proportion of the prescribed diets (between 30–40%) consumed on-site under direct supervision of the research staff [23,24,25,26,27,28]. Subjects included in the analyses were also in weight stable conditions throughout the various isoenergetic protocols. Changes in main cardiometabolic outcomes (mostly plasma lipids) in the trials were consistent with expected changes from other studies in the literature [34,35,36]. Finally, changes in plasma fatty acid profiles were also consistent with the dietary intervention [26]. Post- vs. pre-intervention differences in body weight were examined to further confirm body weight stability and hence isoenergetic feeding conditions. Based on the post- vs. pre-intervention body weight difference of all participants, an arbitrary cut-off of ±1.5 SD (0 ± 1.85 kg) change in body weight was chosen to exclude subjects with a large body weight variation after the intervention. A change within ±1.85 kg most likely reflects normal day-to-day variation in body weight, of which most is due to body water fluctuation [37].

### 2.5. Statistical Analyses

The statistical software package SAS^®^ Studio (v3.6, Cary, NC, USA) was used for all analyses. Extreme values of rEI were excluded on the basis of the Outlier Labeling Rule [38]. Outliers are individual values above Q3 + 2 × (Q3 − Q1) or below Q1 − 2 × (Q3 − Q1) where Q1 and Q3 represent the 25th and 75th percentiles of the rEI distribution, respectively. Baseline characteristics of the participants were compared using two-sided Student *t* tests and chi-square tests, where appropriate.

Mean rEI and mER were compared using MIXED models with self-report flag (indicator variable for rEI or mER), age, sex, body mass index (BMI), ethnicity, trial and post vs. pre-intervention body weight difference as fixed effects, and subject as a random effect. Potential statistical differences between the IA- and WEB-FFQ were assessed with addition of the interaction term FFQ method (IA- or WEB-FFQ) × self-report flag to the MIXED models. Spearman correlations (r_s_) were used to examine the association between rEI and mER with adjustment for age, sex, BMI, ethnicity, trial and post vs. pre-intervention body weight.

Participants were also classified as under-reporters, accurate reporters, or over-reporters on the basis of their ratio of rEI to mER (i.e., a ratio of 1.00 would indicate exact correspondence between both measures). Confidence limits (CL) were calculated around the rEI:mER ratio based on the coefficient of variation (CV) for rEI and mER to account for measurement errors and normal variation in energy expenditure:

(1) $$95\%\;CL=\pm \;2\;\times \sqrt{\left(\frac{C{V_{rEI}}^{2}}{d}+C{V_{mER}}^{2}\right)}.$$

The CV_rEI_ (29.3%) is the within-individual CV in rEI obtained from the WEB-FFQ [15]. Repeated measurement data for the IA-FFQ were unavailable and the same CV_rEI_ was used for both FFQs. The CV_rEI_ was subsequently divided by the number of days (*d*) recalled by the FFQs (i.e., 30 days). The CV_mER_ is obtained from regression equations of doubly labelled water studies and corresponds to measurement error and variation in energy expenditure (i.e., 9.1%) over a time span of 8 weeks [39]. This specific time span was chosen to account for the length of both the dietary intervention and the run-in period of all trials in the present study, as rEI and mER were not measured concurrently. A multiplicative factor of 2 was applied to the combined CV to obtain 95% confidence limits. Thus, individuals were classified as under-reporters or over-reporters if their rEI:mER ratio was below 0.79 or above 1.21, respectively. 

Log-binomial regression models were used to assess the association between BMI and sex and the likelihood of under-reporting. Covariates included in the adjusted models, where appropriate, were sex, BMI, ethnicity, trial and post vs. pre-intervention body weight difference. A two-sided alpha level of less than 0.05 was used to assess statistical significance.

## 3. Results

### 3.1. Participants

**Figure 1:**
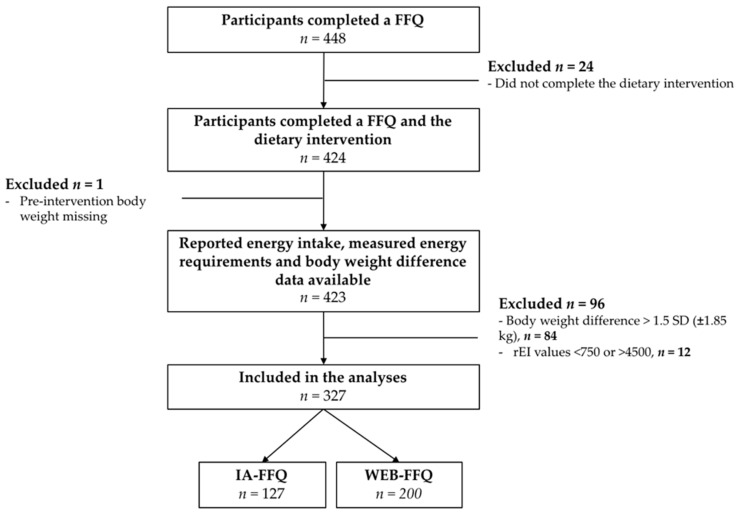
Flow chart of participants. FFQ: food frequency questionnaire; IA: interviewer-administered; rEI: reported energy intake.

Data from a total of 448 men and women were considered for this study. Twenty-four were excluded because they did not complete the first phase of the feeding trials, one participant was excluded because pre-intervention body weight was missing, 12 participants were considered outliers on the basis of their rEI (*n* = 5 for the WEB-FFQ and *n* = 7 for the IA-FFQ) and 84 participants were excluded because of a post- vs. pre-intervention body weight difference greater than ± 1.85 kg (*n* = 54 for the WEB-FFQ and *n* = 30 for the IA-FFQ; Figure 1).

Characteristics of the participants included in the analyses are presented for the IA-FFQ (*n* = 127) and WEB-FFQ (*n* = 200) in Table 1. Participants in the IA-FFQ group were slightly younger, had a lower body weight, waist circumference, and BMI, and included more women than participants in the WEB-FFQ group (all *p* values ≤ 0.02). The median (interquartile range) time for completion of the WEB-FFQ was 42.9 (34.0–59.3) min. Mean post- vs. pre-intervention body weight difference was −0.4 kg (95%CI, −0.6 to −0.3) in men and −0.6 kg (95%CI, −0.8 to −0.5) in women (both *p* values < 0.0001), which is within expected range (Table S1).

### 3.2. Reported Energy Intake Compared with Measured Energy Requirements

Mean differences between rEI and mER and rEI:mER ratios are presented by FFQ method and subgroups in Table 2. Results were similar either expressed as the absolute (in kcal) or relative (in %) difference between rEI and mER for both FFQs in all subgroups. Among all participants, the rEI derived from the IA-FFQ was significantly lower than the mER, by −229 kcal (95% CI, −324 to −133; *p* < 0.0001). The rEI derived from the WEB-FFQ was also significantly lower than mER (−166 kcal; 95% CI, −292 to −39; *p* < 0.0001). The mean differences between rEI and mER were similar between FFQs (*p* = 0.62). The IA-FFQ underestimated mean mER in men and women, as well as in non-obese and obese participants. The WEB-FFQ underestimated mean mER only in men and in obese individuals. Analyses stratified by sex and body weight classification revealed similar rEI to mER differences between the IA- and the WEB-FFQ (all *p* values > 0.30).

Spearman correlations between rEI and mER are presented in Table 2. Among all participants, the correlation was stronger with the IA-FFQ (r_s_ = 0.50; *p* < 0.0001) than with the WEB-FFQ (r_s_ = 0.34, *p* < 0.0001). In men, the correlation between rEI and mER was significant with the WEB-FFQ (r_s_ = 0.40; *p* = 0.0001), but not the IA-FFQ (r_s_ = 0.23; *p* = 0.12). Inversely, in women, the correlation between the rEI and mER was significant with the IA-FFQ (r_s_ = 0.63; *p* < 0.0001), but not with the WEB-FFQ (r_s_ = 0.20; *p* = 0.06).

### 3.3. Under-Reporting and Over-Reporting

**Figure 2:**
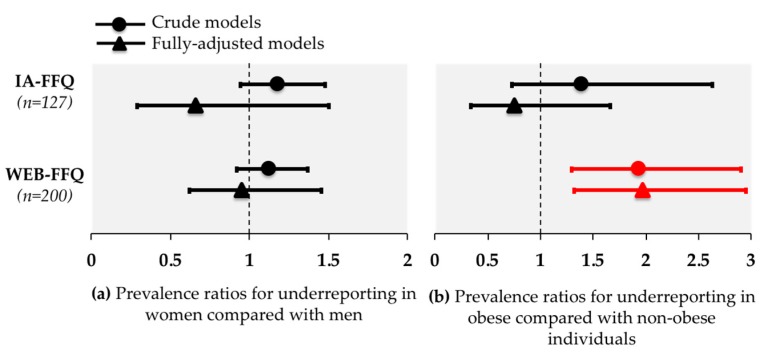
Log-binomial regression analysis showing prevalence ratios with 95% CI for under-reporting in: (**a**) women vs. men and; (**b**) in obese vs. non-obese individuals. Crude models are shown with circles and adjusted models are shown with triangles. Under-reporters are individuals of which their corresponding rEI:mER ratio is below 0.79. CI: confidence interval; FFQ: food frequency questionnaire; IA: interviewer-administered.

Prevalence and likelihood of under-reporting and over-reporting are shown in Table 3 and Figure 2 respectively. Among all participants, under-reporting was more prevalent with the WEB-FFQ than with the IA-FFQ (34.5% vs. 26.0%) but the difference did not reach statistical significance. The prevalence of under-reporting among obese participants was similar with the WEB-FFQ and the IA-FFQ (46.7% vs. 33.3%; *p* = 0.24) and also among non-obese participants (24.1% vs. 24.0% respectively). Obese individuals were more likely to under-report rEI than non-obese individuals with the WEB-FFQ (prevalence ratio, 1.97; 95% CI, 1.32 to 2.95), but not with the IA-FFQ (prevalence ratio, 0.75; 95% CI, 0.34 to 1.66; Figure 2). The prevalence of under-reporting was similar between the WEB-FFQ and the IA-FFQ among women (30.4% vs. 21.1%, respectively) and men (38.0% vs. 33.3%, respectively). Data presented in Figure 2 suggest that women were similarly likely to under-report rEI compared with men with both FFQs (IA-FFQ: prevalence ratio, 0.66; 95% CI, 0.29 to 1.50; WEB-FFQ: prevalence ratio, 0.95; 95% CI, 0.62 to 1.45). Finally, over-reporting was more prevalent with the WEB-FFQ than the IA-FFQ among all participants (*p* = 0.0005), while subgroup differences were statistically significant only in non-obese participants (Table 3).

## 4. Discussion

The aim of this study was to compare the accuracy of IA- and WEB-FFQ to assess an objective measure of energy requirements. Consistent with previous investigations [11], we found that both FFQs resulted in significant under-reporting of mER by −11.0% (WEB-FFQ) and −9.5% (IA-FFQ). In general, and contrary to our hypothesis, results indicated that the IA-FFQ performs slightly better than the WEB-FFQ in attenuating the prevalence of under-reporting and over-reporting in most subgroups based on sex and body weight classification.

Web-based tools such as the WEB-FFQ are being increasingly used in research for several reasons, including greater efficiency in administration process and facilitated data management [15,16,17]. However, studies that have compared the accuracy of traditional IA- and WEB-FFQs in predicting energy and nutrient intake are scarce to date. Park et al. [40] have recently conducted a large study comparing self-reported intakes using self-administered web-based instruments, including a FFQ, against recovery biomarkers. Energy was significantly under-reported by −29 to −34% on average compared with the doubly labeled water technique, the gold standard reference. The degree of under-reporting in the study by Park et al. was greater than in the current study (−11% for the WEB-FFQ), possibly due to different methodologies. Nonetheless, the results by Park et al. are consistent with results in the present study revealing systematic error when estimating energy intakes with a WEB-FFQ.

Kato et al. [41] compared two self-administered FFQs that differed only by their format (paper- vs. web-based) in their ability to accurately predict energy intake, using weighted 12-day food records as reference for “true” energy intake. Energy intake derived from the WEB-FFQ correlated weakly with energy intake derived from the food records, but the correlation was slightly higher among men than among women (Spearman’s deattenuated correlation coefficients = 0.42 and 0.18, respectively). This observation is somewhat consistent with our results as the correlation between rEI and mER for the WEB-FFQ was significant in men, but not in women. Of note, the use of an objective reference method to assess ER in our study may have yielded weaker correlation between rEI and mER than those observed in this study by Kato et al. [41]. These authors also found that energy intakes derived from the WEB-FFQ were within acceptable limits of agreement in men (Bland-Altman method, 54–178%), and were slightly overestimated in women (Bland-Altman method, 55–220%) compared with the energy intakes derived from food records. Results from the present study showed that women had a similar likelihood to under-report energy intake compared with men when using the WEB-FFQ. The weaker correlation between rEI and mER in women when using the WEB-FFQ may be due to over-reporting being more prevalent in women than in men, which is consistent with the overestimation observed by Kato et al. [41]. Nonetheless, these observations contradict previous IA-24HR data that showed greater under-reporting in women [6]. Future studies should provide additional insight on potential sex-based differences on the accuracy of web-based tools in predicting energy requirements.

Another recent study used the doubly labeled water technique to examine the accuracy with which a WEB-FFQ (i.e., MiniMeal-Q) and a web-based 4 days food record (i.e., Riksmaten method) predict energy intake [42]. Pearson’s correlations between rEI and mER were 0.28 (non-significant) for the WEB-FFQ and 0.40 (*p* < 0.05) for the food records. The WEB-FFQ resulted in a higher prevalence of under-reporters compared with the foods records (57.5% vs. 40%, respectively) and also a higher prevalence of over-reporters (15% vs. 5%, respectively). Although this study compared two web-based dietary assessment tools, the results support that current WEB-FFQ may not be better than other dietary assessment tool to estimate true energy intake. This observation is consistent with results from our study in that the WEB-FFQ produced weaker correlation between rEI and mER and a greater prevalence of under- and over-reporters compared with the IA-FFQ.

The rather large number of participants in this study compared with previous studies along with measured energy requirements are important strengths. Potential limitations also need to be considered. Firstly, the WEB-FFQ and the IA-FFQ have notable differences including the number of food items (greater with the WEB-FFQ) as well as different approaches to present serving sizes (food models vs. digital images), which could explain, at least partly, differences in the accuracy of mER prediction. Secondly, different study participants completed the IA- or WEB-FFQ. Therefore, differences observed in this study may not solely be due to the administration technique *per se* (i.e., IA vs. WEB) but could also reflect differences among study participants, although analyses were adjusted for these differences (i.e., age, sex, BMI, ethnicity). Thirdly, the controlled feeding phases were conducted in free-living conditions and some of the foods and beverages provided may not have been entirely consumed. However, the high self-reported compliance combined with the fact that a large proportion of the foods provided were consumed in the presence of study coordinators, the consistency of the cardiometabolic changes induced by the interventions and analysis of plasma biomarkers suggest that the risk of noncompliance in these studies is low. Finally, the significantly lower post- vs. pre-intervention body weight may suggest insufficient food provision (mER) in the feeding phases, but the weight difference was small and also added as a covariate in the analyses.

## 5. Conclusions

In conclusion, results from this study suggest that an IA-FFQ slightly attenuates the prevalence of under- and over-reporting of mER compared with a WEB-FFQ. Accordingly, the use of the WEB-FFQ resulted in accurate reporting of energy intake in 48% of all participants compared with 68% with the IA-FFQ. Considering the efficiency of web-based questionnaires and the importance of dietary assessment for population-based nutrition studies, our results support the urge to increase the quality of web-based dietary assessment tools and to further develop objective and innovative assessment techniques. Future studies should also examine if specific foods or nutrients are more likely to be under- or over-reported in web-based compared with traditional tools. The use of metabolomics and passive measure of one’s food intake through digital imaging and video also have the potential to improve our ability to assess dietary intake [17,43].

## Figures and Tables

**Table 1:** Characteristics of the 327 men and women included in the analyses ^1^.

	IA-FFQ	WEB-FFQ	*p* ^2^
	*N* = 127	*N* = 200	
Sex, *n* (%)			0.01
Men	51 (40.2)	108 (54.0)	
Women	76 (59.8)	92 (46.0)	
Ethnicity, *n* (%)			0.01
Caucasian	121 (95.3)	172 (86.0)	
Other	6 (4.7)	28 (14.0)	
Age ^3^, mean (SD) years	40.9 (16.8)	44.5 (15.4)	0.04
19–34, *n* (%)	58 (45.7)	67 (33.5)	
35–49, *n* (%)	14 (11.0)	44 (22.0)	
50–70, *n* (%)	55 (43.3)	89 (44.5)	
Time to completion, minutes	-	42.9 (34.0 to 59.3)	
Body weight, mean (SD) kg	72.8 (16.6)	84.6 (15.8)	<0.0001
Body mass index, mean (SD) kg/m^2^	25.8 (5.4)	29.7 (4.4)	<0.0001
Normal, *n* (%)	69 (54.3)	27 (13.5)	
Overweight, *n* (%)	31 (24.4)	81 (40.5)	
Obese, *n* (%)	27 (21.3)	92 (46.0)	
Waist circumference, mean (SD) cm	88.4 (14.8)	100.6 (11.8)	<0.0001

^1^ Values are means (SD) for continuous variables except for time to completion which is median (interquartile range). FFQ: food frequency questionnaire; IA: interviewer-administered. ^2^ *p* values indicate differences between the IA-FFQ and the WEB-FFQ, determined by Student’s *t* test or Chi-squared test. ^3^ Analyses were performed on log-transformed data.

**Table 2:** Comparison of reported energy intake (rEI) with measured energy requirement (mER) for maintenance of body weight during a controlled feeding phase of 4 to 6 weeks ^1^.

	*n*	rEI, kcal	mER, kcal	∆ rEI-mER, kcal	∆ rEI-mER, % *	Spearman CC
IA-FFQ						
All	127	2413 ± 602	2642 ± 558	−229 (−324 to −133) †	−9.5 (−12.7 to −6.1)	0.50 ‡
Sex						
Men	51	2744 ± 605	3161 ± 467	−417 (−600 to −234) †	−14.3 (−19.6 to −8.6)	0.23
Women	76	2191 ± 491	2294 ± 265	−102 (−197 to −8) †	−6.1 (−9.9 to −2.0)	0.63 ‡
BMI						
Non-obese	100	2415 ± 610	2594 ± 546	−179 (−282 to −76) †	−7.8 (−11.5 to −4.0)	0.51 ‡
Obese	27	2409 ± 582	2822 ± 573	−413 (−649 to −176) †	−15.3 (−21.9 to −8.1)	0.13
WEB-FFQ						
All	200	2519 ± 962	2684 ± 536	−166 (−292 to −39) †	−11.0 (−15.4 to −6.4)	0.34 ‡
Sex						
Men	108	2764 ± 991	3056 ± 414	−292 (−469 to −116) †	−14.9 (−20.5 to −8.9)	0.40 ‡
Women	92	2231 ± 845	2248 ± 265	−17 (−198 to 163)	−6.3 (−13.1 to 1.1)	0.20
BMI						
Non-obese	108	2583 ± 1021	2543 ± 520	40 (−132 to 212)	−4.1 (−10.4 to 2.7)	0.39 ‡
Obese	92	2443 ± 888	2850 ± 508	−407 (−585 to −230) †	−18.5 (−24.3 to −12.3)	0.27 ‡

^1^ Values are means (SD) or means (95% CI). BMI: body mass index; CC: correlation coefficient; FFQ: food frequency questionnaire; IA: interviewer-administered; mER: measured energy requirement; rEI: reported energy intake; ∆: delta. * Mean percentage differences between rEI and mER were calculated as 100× exponential (mean of log rEI − mean log mER value) – 100; † Indicates a significant difference with mean rEI as determined by mixed models, *p* < 0.05. Analyses were performed on log-transformed data. ‡ Indicates a significant correlation, *p* < 0.05.

**Table 3:** Prevalence of under- and over-reporting of energy intake according to agreement with measured energy requirements ^1^.

	FFQ Method	*n*	Under-Reporters	Accurate Reporters	Over-Reporters	p *
All	IA	127	26.0 (18.6 to 34.5)	67.7 (58.9 to 75.7)	6.3 (2.8 to 12.0)	0.0005
	WEB	200	34.5 (27.9 to 41.5)	48.0 (40.9 to 55.2)	17.5 (12.5 to 23.5)	
Sex						
Men	IA	51	33.3 (20.8 to 47.9)	60.8 (46.1 to 74.2)	5.9 (1.2 to 16.2)	0.12
	WEB	108	38.0 (28.8 to 47.8)	46.3 (36.7 to 56.2)	15.7 (9.5 to 24.0)	
Women	IA	76	21.1 (12.5 to 31.9)	72.4 (60.9 to 82.0)	6.6 (2.2 to 14.7)	0.0063
	WEB	92	30.4 (21.3 to 40.9)	50.0 (39.4 to 60.6)	19.6 (12.0 to 29.2)	
BMI						
Non-obese	IA	100	24.0 (16.0 to 33.6)	69.0 (59.0 to 77.9)	7.0 (2.9 to 13.9)	0.0019
	WEB	108	24.1 (16.4 to 33.3)	51.9 (42.0 to 61.6)	24.1 (16.4 to 33.3)	
Obese	IA	27	33.3 (16.5 to 54.0)	63.0 (42.4 to 80.6)	3.7 (0.1 to 19.0)	0.24
	WEB	92	46.7 (36.3 to 57.4)	43.5 (33.2 to 54.2)	9.8 (4.6 to 17.8)	

^1^ Values are percentages (95% CI). Accurate reporters are individuals of which their corresponding rEI:mER ratio are within the 95% confidence limits of an agreement ratio of 1.00. Under-reporters and over-reporters had a ratio below 0.79 and above 1.21, respectively. BMI: body mass index; CI: confidence intervals; FFQ: food frequency questionnaire; IA: interviewer-administered; mER: measured energy requirements; rEI: self-reported energy intake. * *p* values indicate at least one significant difference between the IA-FFQ and the WEB-FFQ as determined by the Chi-squared test.

## Supplementary Materials

The following are available online at http://www.mdpi.com/2072-6643/10/9/1292/s1. Table S1: Anthropometric characteristics before and after controlled feeding phases of 4 to 6 weeks in men and women.
