# Supplementary material for: Comparing Interviewer-Administered and Web-Based Food Frequency Questionnaires to Predict Energy Requirements in Adults
Source: Nutrients. 2018 Sep 12;10(9):1292. doi: 10.3390/nu10091292 (PMC6165380; doi:10.3390/nu10091292)
Supplement: Supplementary file 1 [file nutrients-10-01292-s001.pdf]

Supplemental table

Table S1. Anthropometric characteristics before and after controlled feeding phases of 4 to 6 weeks in men and women

	Controlled feeding		Difference (95%CI)	*P**
	Pre	Post		
**Men** (*n*=159)				
Body weight, *kg*	89.1 ± 15.6	88.7 ± 15.4	-0.4 (-0.6, -0.3)	<0.0001
Body mass index, kg/m^2	29.2 ± 5.0	29.0 ± 4.9	-0.1 (-0.2, -0.1)	<0.0001
Waist circumference, *cm*	101.0 ± 14.8	100.5 ± 14.7	-0.5 (-0.8, -0.2)	0.0005
**Women** (*n*=168)				
Body weight, *kg*	71.7 ± 13.7	71.1 ± 13.6	-0.6 (-0.8, -0.5)	<0.0001
Body mass index, kg/m^2	27.4 ± 5.1	27.2 ± 5.1	-0.2 (-0.3, -0.2)	<0.0001
Waist circumference, *cm*	91.1 ± 13.0	90.1 ± 13.3	-1.0 (-1.5, -0.6)	<0.0001

Values are means ± SDs unless otherwise indicated.
* Differences between post- and pre-intervention values, as determined by mixed models.
